# Sleep deprivation: a comprehensive review of multisystem impacts, underlying mechanisms, and emerging interventions

**DOI:** 10.3389/fneur.2026.1819968

**Published:** 2026-06-09

**Authors:** Yong-Zheng Fan, Guo-Dong Liu, An-Na Zhang, Yu Wang, Yu-Qing Cheng

**Affiliations:** The 991st Hospital of Joint Logistic Support Force of People’s Liberation Army, Xiangyang, Hubei, China

**Keywords:** caffeine, interventions, mechanisms, physical therapies, review, sleep deprivation

## Abstract

Sleep deprivation (SD) has become a pervasive public health issue with significant ramifications for physiological and cognitive function. This review aims to provide a comprehensive synthesis of the detrimental health effects of SD, explore the underlying pathophysiological mechanisms, and evaluate current and emerging intervention strategies. We conducted a narrative review of the literature, synthesizing findings from epidemiological, clinical, and preclinical studies. The results indicate that SD profoundly disrupts the function of multiple organ systems, including the nervous, cardiovascular, respiratory, digestive, immune, and endocrine systems. At the mechanistic level, SD induces neuroinflammation, disrupts the intestinal barrier and gut microbiota, impairs hippocampal structure and function, and alters synaptic plasticity. Interventions span pharmacological approaches—from traditional central nervous system stimulants (e.g., caffeine, amphetamines, and modafinil) and sedative-hypnotics to novel therapies such as orexin receptor antagonists and gut microbiota modulators—and non-pharmacological strategies, including napping, physical therapies (e.g., transcranial magnetic stimulation, and light therapy), exercise, cognitive behavioral therapy, and dietary modifications. In conclusion, the evidence highlights the crucial role of adequate sleep in maintaining health. A multifaceted approach combining pharmacological and non-pharmacological interventions, tailored to individual and population-specific needs, holds the greatest promise for mitigating the adverse effects of SD. Future research should focus on personalized interventions and leverage advanced technologies to elucidate the pathogenesis of SD further and develop more effective countermeasures.

## 1 Introduction

Normal sleep is essential for immune enhancement and health maintenance (1). According to the National Sleep Foundation, adults should get no less than 7 hrs of sleep per day, and athletes may need more sleep (2). But sleep deprivation (SD) is becoming more common in modern society, with more than a third of adults reporting SD in the United States and similar patterns in other countries (3). However, only 10% of people seek medical treatment due to SD symptoms. With the acceleration of work pace, the increased use of electronic devices, and other factors, the number of people suffering from SD continues to rise.

SD is divided into total sleep deprivation (TSD) and partial sleep deprivation (PSD) (4, 5), as well as acute sleep deprivation (ASD) and chronic sleep deprivation (CSD) (6). SD not only exacerbates fatigue levels and reduces job performance, alertness, and health, but also negatively affects mood, cognitive performance, and motor function (7), leading to changes in disease susceptibility or behavior, increasing the prevalence of chronic diseases such as obesity, diabetes, and hypertension, and causing multiple diseases, even cancer and death (8).

SD is usually caused by keeping awake while taking stimulants, health problems, and choices of work, study or lifestyle, so it is very important to scientifically formulate reliable prevention or countermeasures against the potential harm induced by SD. This review aims to explain the diseases and mechanisms related to SD, and to focus on intervention strategies to provide new perspectives and enlightenment for improving sleep quality, protecting cognitive function, and delaying the progression of related diseases.

## 2 Methodology

This narrative review was conducted by searching for literature published in PubMed-based literature up to February 2026. The search terms included combinations of the following keywords: “sleep deprivation,” “total sleep deprivation,” “partial sleep deprivation,” “chronic sleep restriction,” “circadian rhythm,” “clock genes,” “neuroinflammation,” “gut microbiota,” “interventions,” “caffeine,” “modafinil,” “orexin receptor antagonists,” “transcranial magnetic stimulation,” and “cognitive behavioral therapy.” We included original research articles, systematic reviews, and meta-analyses from both preclinical (animal) and clinical (human) studies. Reference lists of retrieved articles were also screened to identify additional relevant studies. The selection prioritized studies that provided mechanistic insights or evaluated intervention efficacy in the context of sleep deprivation.

## 3 Classifications of SD

SD is not a uniform condition; its physiological and cognitive consequences vary substantially depending on the duration, timing, and sleep stages affected. A clear taxonomy is essential for interpreting the mechanistic and clinical literature. Based on established frameworks (4–6, 9, 10), SD can be categorized into the following paradigms:

*TSD*: Complete absence of sleep for a defined period, typically ≥24 h. TSD is commonly used in experimental settings to study acute effects on cognition, alertness, and neuroinflammation (11).

*PSD*: Insufficient sleep duration (usually ≤6 h per night) without complete elimination of sleep. PSD more closely resembles real-world sleep restriction (12, 13).

*REM-SD (RSD)*: Selective loss of rapid eye movement (REM) sleep while preserving non-REM sleep. This paradigm is used to isolate the contributions of REM sleep to memory consolidation and emotional regulation (14, 15).

*ASD*: Short-term deprivation lasting less than 48 h, often used to study immediate cognitive and physiological responses (16).

*Chronic sleep deprivation or chronic sleep restriction (CSD/CSR)*: Sustained reduction in sleep duration over multiple days or weeks (e.g., ≤6 h/night for ≥7 days). This paradigm models the cumulative effects of insufficient sleep commonly seen in shift workers and individuals with lifestyle-related sleep loss (17).

Taking these factors into account, the objective of this paper is to conduct a critical and systematic literature review, based on experimental models of SD in human subjects or animals, we specify the SD paradigm referenced for each finding to allow appropriate interpretation of the evidence. Table 1 summarizes the defining features and representative outcomes associated with each SD type.

**Table 1:** Classifications of SD and associated outcomes.

SD type	Definition	Common models	Key outcomes	References
TSD	No sleep ≥24 h	Human laboratory studies; rodent forced locomotion	Impaired attention, working memory deficits, elevated inflammatory markers	(6, 72)
PSD	≤6 h sleep/night	Human laboratory studies; Human self-report; sleep restriction protocols	Increases formation of false memory, metabolic dysregulation	(54, 134)
RSD	Selective REM loss	Multiplatform water environment technique(rodent)	Memory consolidation deficits, altered emotional reactivity	(40, 87, 135)
ASD	≤48 h sustained wakefulness	Human laboratory; acute restraint (rodent)	Alertness decline, increased sleep pressure, transient neuroinflammation	(19, 21, 136)
CSD	≤6 h/night for ≥7 days	Chronic sleep restriction protocols	Persistent cognitive deficits, gut dysbiosis	(22, 63)

## 4 Negative health effects of SD

### 4.1 Effects on the nervous system

SD can cause fluctuations in thalamic activity, changes in synaptic plasticity, amygdala and hippocampus activity, resulting in unbalanced brain stimulation, affecting attention, working memory, alertness, judgment, decision-making, and emotional variability (18). Acute SD reduces neural alertness and responsiveness to external stimuli (19, 20). After partial SD, even for one night, alertness will be affected, and executive function and inhibitory control ability will also decline (21). Acute SD for about 4.5 h can produce negative effects on athletes ‘alertness. Adolescents who sleep less than 5 h a day for 4 consecutive nights can also produce significant alertness damage, which is manifested in significantly prolonged psychomotor alertness reaction time and significantly reduced accuracy (22), and impairs working memory capacity (23), Affects an individual’s anxiety symptoms and emotional response to events, increases the risk of depression (24) and susceptibility to neurological disorders such as Alzheimer’s (25).

### 4.2 Effects on the cardiovascular system

SD will stimulate the activity of the sympathetic nerve and reduce the activity of the parasympathetic nerve, disrupting the dynamic balance between the sympathetic and parasympathetic nerves, and weakening the protective effect of the autonomic nervous system (26), reducing the blood flow velocity in the coronary arteries (27), and inducing cardiovascular diseases such as hypertension, atherosclerosis, arrhythmia and myocardial infarction (28). SD is associated with an increased risk of cardiovascular diseases and the formation of atherosclerotic plaques. Vascular endothelial dysfunction is a precursor of atherosclerosis and cardiovascular diseases. Cherubini et al. (29) demonstrated that SD leads to an increase in reactive oxygen species (ROS), nitric oxide (NO), superoxide dismutase (SOD), and catalase (CAT) levels, reduces exosomal miR-182-5p, which promotes endothelial inflammation and the formation of atherosclerosis (30); impairs the endothelial function of microvessels and large vessels; reduces ejection fraction; and increases the incidence and mortality of cardiovascular diseases (CVD).

### 4.3 Effects on the respiratory system

The relationship between sleep disturbances and respiratory disease is bidirectional, with underlying respiratory conditions leading to sleep fragmentation, and insufficient sleep further exacerbating respiratory dysfunction. It is important to distinguish obstructive sleep apnea syndrome (OSAS) from classical SD: Obstructive sleep apnea syndrome is a very common disorder. During sleep, the upper airway repeatedly collapses, resulting in partial or complete obstruction of the airway, causing intermittent hypoxia and sleep interruption. These conditions disrupt the integrity of sleep, leading to chronic PSD and accompanied by destructive metabolic sequelae (31–33). Nevertheless, the two conditions frequently co-occur and may share overlapping pathophysiological mechanisms. A study conducted by MD de Kruif et al. shows that patients with obstructive sleep apnea syndrome (OSAS) have an infection risk of COVID-19 that is nearly 8 times that of their peers. Among COVID-19 patients, OSAS is associated with a higher risk of hospitalization, and the risk of respiratory failure is approximately 2 times higher (34). Christophe Rault et al. assessed the effects of SD on respiratory motor output and inspiratory endurance. Overnight SD reduces respiratory motor output by changing cerebral cortex components in healthy subjects, thus halving inspiratory endurance, which may cause severe brain dysfunction and may lead to respiratory failure (35). A prospective cohort study confirmed that patients with asthma who were sleep deprived had significantly lower total IgE and FeNO levels, higher airway inflammation, and elevated levels of IL-6 and TNF-*α* in sputum, resulting in a significantly increased risk of asthma (36).

### 4.4 Effects on the gastrointestinal system

After SD for 3 months, Zhang et al. significantly increased the number of throat reflux attacks in prone rats (37), suggesting that there is a bidirectional relationship between gastroesophageal reflux disease (GERD) and sleep. Night reflux leads to SD, and SD itself may aggravate the occurrence of GERD by enhancing the perception of intraesophageal stimuli (38, 39). SD in REM sleep stage will increase and lead to irritable bowel syndrome (IBS) gastrointestinal motor symptoms (40). Fang et al. (41) conducted a follow-up of 1,420 elderly patients with peptic ulcer who had eradicated *Helicobacter pylori* infection for up to 36 months. They found that patients with poor sleep quality were more likely to have peptic ulcer recurrence, suggesting that SD is a risk factor for peptic ulcer incidence and recurrence. SD can induce oxidative stress, cause nonalcoholic fatty liver disease (42), and thus adversely affect the digestive system. In addition, SD impairs intracellular lipolysis of KynA, leading to the accumulation of lipid droplets in colon cancer cells and promoting liver metastasis of colon cancer (43).

### 4.5 Effects on the immune system

SD disrupts immune function, reduces host defense against infection and inflammatory damage (44), and even reduces immune response to vaccines (45). Acute SD can trigger neutrophil hyperactivation in mice, resulting in abnormal peripheral accumulation and systemic cytokine storm (46). CSD reduces melatonin levels, elevates pro-inflammatory cytokines (such as CRP, IL-6, and TNF-*α*), and decreases neutrophil levels (47, 48), increases oxidative stress and decreased activity including natural killer (NK) cells and CD4 + lymphocytes (49), leading to increased risk of chronic inflammatory states and infectious/inflammatory lesions in tumors, autoimmune and neurodegenerative diseases (50). SD has been reported to cause inflammatory diseases in young people and infectious diseases in the elderly, and may trigger autoimmune diseases such as systemic lupus erythematosus, scleroderma, or rheumatoid arthritis (51, 52).

### 4.6 Effects on the endocrine system

Epidemiological evidence confirms an association between SD and endocrine metabolic disorders, particularly overweight/obesity (53). Insufficient sleep consistently increases food intake, potentially raising the risk of weight gain and obesity, while adequate sleep facilitates weight loss. To maintain wakefulness during SD, healthy adult volunteers need to increase food intake to provide the required energy (54), switching from SD to adequate/restorative sleep reduces energy intake, particularly fat and carbohydrate components, favoring weight loss (55), This suggests that sleep regulates body mass index (BMI) by influencing the balance between energy expenditure and intake; further exploring and improving this balance could aid in intervening against overweight or obesity (56). Importantly, SD causes alterations in glucose metabolism and levels of hormones like leptin and ghrelin, reducing an individual’s ability to control blood glucose after SD (57) and increasing the risk of type 2 diabetes (58).

## 5 Mechanisms underlying SD-related disorders

### 5.1 Circadian disruption as a Core mechanism

SD and circadian misalignment are deeply intertwined but mechanistically distinct. The circadian system, governed by the suprachiasmatic nucleus (SCN) in the hypothalamus, coordinates daily rhythms in physiology and behavior through transcriptional feedback loops involving core clock genes—including CLOCK, BMAL1, *PER1/2/3*, *CRY1/2*, and REV-ERBα/*β* (59–62). These molecular clocks operate not only in the SCN but also in peripheral tissues such as the gut, liver, and immune cells, where they regulate local metabolic and inflammatory processes. SD disrupts circadian rhythms through two interrelated mechanisms: (i) increased sleep homeostatic pressure (Process S), which drives the need for recovery sleep, and (ii) circadian phase disruption (Process C), which desynchronizes internal biological rhythms from external cues (6). Many effects traditionally attributed solely to sleep loss—such as gut microbiota dysbiosis, HPA axis dysregulation, and metabolic impairment—are now recognized to be mediated at least in part through circadian disruption (63, 64).

For instance, Thaiss et al. (65) demonstrated that the gut microbiota exhibits diurnal oscillations in composition and function, driven by host circadian rhythms. These oscillations are abolished by sleep disruption and can be transferred via fecal transplantation, indicating transkingdom control of circadian physiology. Similarly, Leone et al. (66) showed that circadian disruption alters gut microbial composition and promotes metabolic dysfunction. In the context of SD, the loss of circadian rhythmicity in microbial short-chain fatty acid (SCFA) production and tryptophan metabolism contributes to systemic inflammation and neurobehavioral deficits (59, 67). Throughout the following sections, we explicitly address whether the effects described are driven by sleep homeostatic pressure (Process S), circadian phase disruption (Process C), or their interaction. Where evidence permits, we distinguish between outcomes attributable to sleep loss per seversus those resulting from circadian misalignment.

### 5.2 Neuroinflammation

SD acts as a stressor, triggering inflammatory responses and neuropsychiatric disorders (68) (rodent and human studies). It leads to overactivation of the hypothalamic–pituitary–adrenal (HPA) axis and sympathetic nervous system pathways, increasing pro-inflammatory cytokine activity (68). When excessive or sustained, this inflammatory response can cause neuronal damage and neural network dysfunction (69). Studies in rodents show that SD indirectly induces neuroinflammation by activating microglia and altering astrocyte-neuron signaling (70). Human studies have confirmed elevated circulating levels of IL-6 and TNF-*α* following sleep restriction (47, 48). It will also destroy the homeostasis of neurotransmitters, destroy the levels and functions of various important neurotransmitters, including dopamine, 5-hydroxytryptamine, acetylcholine, glutamate, and GABA, destroy the blood–brain barrier (71), increase its permeability, and allow peripheral inflammatory factors and harmful substances to enter the CNS (72). These all aggravate neuroinflammation. Neuroinflammation and neurotransmitter imbalance reinforce each other in a vicious cycle, leading to secondary brain tissue damage, cognitive decline, and other neurological diseases (3).

### 5.3 The bidirectional microbiota–sleep axis

The relationship between SD and gut microbiota is bidirectional and mechanistically complex. SD disrupts the composition and rhythmicity of gut microbiota, leading to dysbiosis characterized by expansion of Proteobacteria, loss of beneficial microbes (e.g., *Akkermansia muciniphila*, Lactobacillus, Bifidobacterium), and impaired metabolic pathways (73, 74). Conversely, microbiota-derived metabolites influence sleep regulation through multiple pathways, forming a reciprocal gut–brain axis that perpetuates sleep disturbances. Microbial metabolites and sleep regulation. Short-chain fatty acids (SCFAs), particularly butyrate, are key microbial metabolites that influence sleep. Butyrate promotes sleep by modulating orexin neuron activity in the lateral hypothalamus (67). SD reduces butyrate levels in both fecal content and the hypothalamus, and oral butyrate supplementation rescues sleep disturbances in germ-free mice (67). The tryptophan–serotonin–melatonin axis represents another critical pathway: gut microbiota regulate tryptophan metabolism, influencing serotonin (5-HT) synthesis in the intestine, which in turn affects melatonin production and circadian entrainment (59, 75). Disruption of this axis underlies the sleep disturbances observed in dysbiotic states. Circadian rhythmicity of microbiota and intestinal barrier disruption. The gut microbiota exhibits diurnal oscillations in composition and function, driven by host circadian rhythms via core clock genes (59). Thaiss et al. (65) demonstrated that these oscillations are abolished by sleep disruption, leading to loss of rhythmicity in microbial SCFA production and increased susceptibility to metabolic dysfunction. SD also impairs the intestinal mucosal barrier by reducing the expression of tight junction proteins (occludin, claudin-1), decreasing the number of intestinal goblet cells and mucin levels (76), and suppressing colonic expression of the clock factor PER2. This leads to COX-2-mediated dysregulation of arachidonic acid (AA) metabolism, releasing pro-inflammatory mediators and contributing to depression, anxiety, colitis, endocrine metabolic disorders, and cardiovascular diseases (64, 77). The microbiota–taurine–Nr1d1 axis has been identified as a key pathway linking SD to intestinal barrier dysfunction: SD reduces taurine-producing bacteria, leading to downregulation of Nr1d1 and impaired epithelial barrier integrity (63, 78). Therapeutic implications. Given the bidirectional nature of this axis, interventions targeting the gut microbiota—including probiotics, prebiotics, dietary modifications, and fecal microbiota transplantation—hold promise for mitigating SD-induced neurological and metabolic consequences (73, 74). However, most evidence remains preclinical, and human trials are needed to establish efficacy.

### 5.4 Hippocampal impairment

Evidence suggests that SD significantly reduces the density of Ki-67 and DCX immunopositive cells in the subgranular zone of both the dorsal and ventral hippocampus in rats, decreasing hippocampal cell proliferation and neurogenesis in the dentate gyrus (79). Gaine et al. (80) using unbiased deep RNA sequencing, identified 1,146 significantly dysregulated genes in the hippocampus after acute SD. Severe metabolic disturbances occur in the hippocampus involving glycerophospholipids, linoleic acid, alanine, aspartate, glutamate, taurine, hypotaurine, and purines (81). These disturbances impair cAMP and mTOR signaling, as well as IL-17 and p38 MAPK pathways (82, 83), contributing to memory impairments and other neurological disorders.

### 5.5 Impact on synaptic plasticity

Growing evidence links cognitive impairment following SD to alterations in synaptic plasticity (84). Synaptic plasticity, the ability of synapses to change in number, morphology, structure, function, and transmission efficacy in response to internal and external environmental changes (85), involves hundreds of synaptic proteins and intercellular signaling pathways and is considered the neurobiological basis for cognitive functions like learning and memory (86). Studies (87) have found that after REM SD in rats, the pre- and postsynaptic membranes in the hippocampal CA1 region become thinner, synaptic structures blur, gaps disappear or narrow, synaptic interface length shortens, and the number of synaptic vesicles decreases significantly, potentially inducing cognitive deficits. Signaling pathways related to synaptic plasticity are closely interconnected and interact. Research indicates that SD downregulates cAMP response element-binding protein (CREB) and mammalian target of rapamycin (mTOR) signaling. For example, Li et al. (88) showed that 21 days (18 h/day) of SD reduced CREB expression, and Golgi staining revealed significantly decreased dendritic spine density in the DG region of the hippocampus. CREB signaling, due to its critical role as a cellular mechanism of synaptic plasticity, represents a potential therapeutic target for memory disorders (88).mTOR, a protein kinase involved in translational control and persistent synaptic plasticity, when dysregulated, can affect synaptic plasticity and lead to various diseases, including cognitive impairment (89, 90) (Table 2).

**Table 2:** Summary of key mechanisms linking SD to organ-system dysfunction.

Mechanism	Affected systems/Outcomes	Key molecular pathways	Representative evidence (Species)
Neuroinflammation	Nervous system: cognitive impairment, mood disorders, neurodegeneration	Microglial activation, NF-κB, HMGB1/TLR4, pro-inflammatory cytokines (IL-1β, IL-6, TNF-α)	Human (47, 48, 72), Rodent (70)
Circadian disruption	Multiple systems: metabolic, immune, gut	Core clock genes (CLOCK/BMAL1/PER/CRY), SCN, Process S/C interaction	Human (6), Rodent (59–62, 64)
Gut microbiota dysbiosis	Gastrointestinal: barrier dysfunction, colitis; systemic: metabolic, neuroinflammatory, increased permeability, endotoxemia	PER2–COX-2 axis, microbiota–taurine–Nr1d1, SCFA reduction, tryptophan–serotonin–melatonin pathway; Tight junction proteins (occludin, claudin), goblet cell ER stress	Rodent (63, 67, 76–78)
Hippocampal impairment	Cognition: memory deficits, learning impairment	Reduced neurogenesis (Ki-67, DCX), altered transcriptome (1,146 dysregulated genes), glycerophospholipid metabolism	Rodent (79–81)
Synaptic plasticity impairment	Cognition: learning/memory deficits	CREB, mTOR signaling, dendritic spine density reduction	Rodent (87–90)
Oxidative stress	Cardiovascular: endothelial dysfunction, cardiac fibrosis; CNS: neuronal damage	ROS, GSH/GSSG imbalance, GPX4, Sirt1, Gsta3	Rodent (121–125)
HPA Axis dysregulation	Endocrine: cortisol elevation, metabolic dysregulation	Corticosterone, CRH, ACTH	Human (72)

Human studies are primarily observational cohorts or controlled laboratory protocols; rodent studies derive from controlled experimental models.

## 6 Pharmacological interventions

### 6.1 Central nervous system stimulants

#### 6.1.1 Amphetamine

Amphetamine, developed in the 1930s, can maintain confidence, performance, and morale (91). It is a potent psychostimulant believed to sustain alertness by binding to dopaminergic receptors and blocking dopamine reuptake, leading to widespread dopaminergic activity and activation of the brain’s reward centers (92). Numerous studies have demonstrated its efficacy in maintaining alertness and vigilance during SD (93), and it has been successfully used as an anti-fatigue measure in various combat aviation operations. During World War II, air raids on Libya, and the Gulf War, U. S. military personnel were administered dextroamphetamine during flights, showing significant effects against SD (94). However, amphetamine has strong excitatory effects that transiently enhance alertness and reduce fatigue, but its severe addictive potential, dependence, and side effects such as hallucinations, toxic symptoms with repeated use, and aviation medicine-related adverse effects limit its use (95).

#### 6.1.2 Caffeine

Caffeine, an adenosine receptor A2A subtype antagonist, increases sleep latency and promotes wakefulness, maintaining alertness, and is widely used to enhance vigilance and cognitive performance (96). In a placebo-controlled 50-h TSD protocol, administering four 200 mg doses of caffeinated gum during the nocturnal circadian trough reduced subjective sleepiness and produced significant improvements in alertness and procedural decision-making during task performance (97). It also improves driving performance and reaction time in subjects (98). However, reports indicate that daily repeated intake of 300–450 mg caffeine does not improve alertness in rested and sleep-restricted individuals (99), After brief initial benefits, when sleep restriction exceeds three nights, caffeine may even enhance sleepiness and attention deficits compared to placebo (100), Thus, not only dosage and frequency but also significant individual differences affect its efficacy against SD (101). Importantly, caffeine has a half-life of approximately 5–6 h, and consumption later in the day can disrupt circadian rhythms by delaying the evening rise in melatonin and altering subsequent sleep architecture (102). Evidence level: Multiple randomized controlled trials; effect sizes moderate for vigilance improvement; limitations include inter-individual variability and tolerance with repeated use. Researchers have explored various formulations, including sustained-release preparations and nanoparticles (103), for more convenient and faster action suitable for various environments.

#### 6.1.3 Modafinil

Modafinil, a neuroprotective agent and wakefulness-promoting substance classified as a “cognitive enhancer,” is used to treat excessive sleepiness associated with narcolepsy, obstructive sleep apnea, and shift work disorder. It inhibits dopamine reuptake, enhancing dopaminergic signaling in the prefrontal cortex and restoring dopamine levels, thereby improving cognitive impairment. Xu et al. (104) found that 400 mg of modafinil significantly ameliorated the decline in working memory after 36 h of SD. It also maintains alertness, enhances cognitive function, and improves risk judgment in pilots undergoing SD (105). Compared to other central stimulants, modafinil effectively promotes wakefulness and enhances cognition without causing sedative effects, euphoria, or disrupting normal sleep. Long-term use shows no dependence or addiction tendencies (106). However, repeated administration of modafinil may not prevent performance deterioration during prolonged SD and could even lead to overconfidence in one’s cognitive abilities (107), often accompanied by side effects like headache, nausea, and potential for abuse (108). Zhu et al. (109)developed a high-drug-loading microneedle patch for self-administration. Compared to oral tablets, microneedle delivery maintained more prolonged and stable plasma drug concentrations and improved cognitive function in sleep-deprived mice, showing promise for transdermal administration in protecting cognitive function.

### 6.2 Sedative-hypnotics

The American Academy of Sleep Medicine (AASM) suggests that longer SD periods may warrant the use of hypnotic medications, i.e., increasing sleep before a known period of SD (107). Benzodiazepine sedative-hypnotics (e.g., triazolam, temazepam) for treating sleep disorders are used in some countries for shift work or night duties. To minimize residual effects, non-benzodiazepine rapid-onset sedative-hypnotics with shorter half-lives, such as zaleplon or zolpidem, are used for sleep induction. They effectively counter SD by increasing sleep quality and depth, allowing individuals to restore and enhance cognitive performance shortly after awakening with fewer adverse reactions (110). Administering melatonin and melatonergic drugs can shorten sleep latency, increase total sleep time, and improve sleep quality. For example, ramelteon, a melatonin receptor agonist acting on MT1 and MT2 receptors, is FDA-approved for insomnia treatment. Clinical trials demonstrate that ramelteon (8 mg) can effectively shorten the sleep latency period, and it has no significant or clinically relevant residual effects. It also generally increases the total sleep time (objective), sleep latency and sleep quality (objective and subjective) compared with placebo, and has been proven to have no possibility of abuse or dependence (111–113). However, its chronobiotic efficacy for circadian reentrainment is modest compared to appropriately timed exogenous melatonin (112, 114). Evidence level: Multiple randomized controlled trials for sleep onset latency. It and other melatonin agonists (tasimelteon and agomelatine) exhibit chronobiotic properties that may prove particularly helpful in treating circadian rhythm disorders (112, 114).

### 6.3 Orexin receptor antagonists

Reports indicate that SD increases activity in the hypothalamic orexin system and the risk of Alzheimer’s disease (AD) (115). Orexin A (OXA) and Orexin B (OXB) are neuropeptides produced in the lateral hypothalamus that influence the sleep–wake cycle and stress response by interacting with OX1 and OX2 receptors. Yaran Li et al. demonstrated that SD exacerbated learning and memory deficits and increased cerebral *β*-amyloid (Aβ) deposition in AD mice. Intraperitoneal injection of Almorexant, a dual orexin receptor antagonist (DORA), increased total sleep time and reduced cognitive impairment and Aβ deposition in sleep-deprived mice (115). Several dual OXR antagonists (DORAs), such as suvorexant (2014) and daridorexant (2022), have received FDA approval for insomnia treatment. DORA treatment reduces Aβ deposition in the brain, improves synaptic plasticity and circadian rhythms, representing a potential effective drug for treating insomnia and delaying disease progression in AD patients by ameliorating sleep disturbances (116).

### 6.4 Traditional Chinese medicine

Traditional Chinese Medicine (TCM) is characterized by a wide variety of resources and can prevent and treat physical fatigue damage and memory dysfunction caused by insufficient sleep through multi-component, multi-target, and multi-pathway effects (117). Liuwei Anshen Capsule (LAC) alleviates cognitive abnormalities and pathological changes in sleep-deprived rats by regulating apoptosis-related proteins and MAPK signaling pathways (118). Banxia Xiexin Decoction (BXHP) improves circadian rhythm rhythmicity, promotes sleep, and ameliorates anxiety by activating the cAMP-PKA-CREB-circadian rhythm pathway (119). Lonicerae Japonicae Flos (LJF), a commonly used Chinese herb with antiviral, antioxidant, and anti-inflammatory properties, has been shown via polysomnography to reduce sleep latency and increase rapid eye movement (REM) sleep time in mice during recovery from acute SD (120). These studies provide mechanistic insights into the potential therapeutic effects of TCM for SD-related disorders. However, it is important to note that the evidence for TCM interventions is predominantly preclinical, derived from rodent models. While the mechanistic findings—such as regulation of the cAMP-PKA-CREB circadian pathway (119) and anti-inflammatory effects (118)—are promising, translational validation in human populations is required before clinical recommendations can be made. Evidence level: Preclinical *in vivo* rodent studies.

### 6.5 Antioxidants

SD-induced cognitive impairment is associated with oxidative stress damage in the hippocampus. This damage can be reflected by biomarkers, including the activity of antioxidant enzymes (SOD, T-AOC, GPX4, and catalase), the ratio of reduced glutathione (GSH) to oxidized glutathione (GSSG), and levels of lipid peroxidation products like malondialdehyde (MDA) and oxidized glutathione (GSSG) (121). Depletion of GSH and impaired GPX4 activity contribute to the accumulation of lipid peroxides, exacerbating cell death. Vitamin B6 alleviates hippocampal lipid peroxidation in mice subjected to CSD, replenishes intracellular GSH deficiency, and restores GPX4 expression, exerting a protective effect against SD-induced hippocampal damage (122). Soy isoflavones and Vitamin C possess antioxidant properties, reducing MDA levels and preventing cognitive impairment in mice or enhancing stress resistance in rats caused by CSD (123). Cerebrolysin, a neuropeptide used for vascular dementia, reverses the SD-induced decrease in GPX, catalase, GSH, and the GSH/GSSG ratio in the rat hippocampus, normalizing elevated GSSG levels, thereby enhancing stress tolerance and improving memory (124). Alpha-lipoic acid (LA) and N-acetylcysteine (NAC), via Sirt1 and Gsta3 pathways, increase serum GSH and SOD levels, reduce cardiac and serum ROS generation, and lower BNP and MDA levels. They effectively reverse CSD-induced cardiac dysfunction, myocardial hypertrophy, mitochondrial dysfunction, and myocardial fibrosis in mice, preventing heart damage caused by sleep disorders and other cardiovascular diseases (125).

### 6.6 Gut microbiota modulators

Disruption of sleep patterns induces gut microbiota dysbiosis (characterized by expansion of Proteobacteria, loss of beneficial microbes, and impaired metabolic pathways), leading to dysbiosis marked by altered microbial composition and function, which exacerbates neurological diseases. To address these challenges, there is growing interest in therapeutic interventions aimed at restoring gut microbial balance and alleviating neurological symptoms resulting from SD (73). Studies show that extracts of *Salvia miltiorrhiza* and Astragalus membranaceus remodel the gut microbiota composition, protect intestinal and blood–brain barrier function, and significantly improve spatial learning and memory deficits in CSD rat models (126), while alleviating depressive-like symptoms in mice with SD (127). Clinically, probiotics are generally considered safe and well-tolerated in healthy populations. They not only modulate the gut microbiota but also improve sleep quality through the production of beneficial metabolites (75). Compared to other strategies, probiotics show potential in mitigating risks associated with SD due to their favorable safety profile (74). However, the evidence base remains predominantly preclinical. Studies in rodent models demonstrate that probiotics (e.g., *Lactobacillus*) can suppress microglial activation, restore tight junction protein expression, and improve cognitive outcomes following SD (74) Limited human studies suggest that probiotic supplementation may improve subjective sleep quality, but effect sizes are modest and heterogeneity across formulations is high (75). Evidence level: Preclinical (rodent) studies predominate; a small number of human RCTs exist for sleep quality outcomes, but not specifically for SD (128). Effect sizes: Not yet quantified for SD-specific outcomes. Limitations: Variability in probiotic strains, dosages, and treatment durations; lack of standardized outcome measures (74).

## 7 Non-pharmacological interventions

### 7.1 Napping

Napping offers an intuitive advantage over other methods as it engages natural recovery processes. Even minor increases in sleep can reduce the negative impacts of SD. Naps lasting 30 to 50 min serve as a strategy for night shift workers to combat sleepiness, reducing accidents and improving alertness and performance through beneficial restorative effects (human studies) (129). In drivers and healthcare professionals, napping may be as effective as caffeine in reducing fatigue and sleepiness, proving an effective fatigue countermeasure (human RCTs) (130).

### 7.2 Physical therapies

Non-invasive brain stimulation physical therapies, utilizing techniques such as light, sound, electricity, magnetism, and mechanical force, combined with electrophysiology and neuromodulation, are emerging as promising approaches for improving SD. These include light therapy, electroacupuncture, transcranial magnetic stimulation (TMS), deep brain stimulation (DBS), transcutaneous electrical acupoint stimulation (TEAS), and transcranial direct current stimulation (tDCS). Light therapy modulates circadian rhythms by acting on the suprachiasmatic nucleus and inhibiting pineal melatonin secretion, helping to re-establish regular sleep–wake cycles and positively impacting sleep structure in Parkinson’s disease (PD) patients (131). Electroacupuncture reduces hippocampal inflammation and improves hippocampal synaptic plasticity in rats (132), inhibits oxidative stress and autophagy, and significantly ameliorates SD-induced cognitive impairment and spatial memory deficits. High-frequency rTMS during SD positively affects the HPA axis and frontal lobe activation, potentially contributing to long-term relief of cognitive impairment (133), 1 Hz repetitive transcranial magnetic stimulation (rTMS) may ameliorate spatial learning and memory deficits induced by chronic RSD in rats, potentially by downregulating kynurenine 3-monooxygenase (KMO) expression and improving the structure and number of hippocampal synapses (137). Transcutaneous auricular vagus nerve stimulation (taVNS), a safe and effective peripheral neuromodulation technique, can modulate cognitive performance, attention, and arousal. taVNS significantly improves accuracy in the spatial 3-back task and enhances performance in the psychomotor vigilance task (PVT) (138, 139), as well as improving performance in arousal and multitasking. Physical therapy techniques are becoming effective treatments for SD. However, objective evaluation of their modulatory effects and optimal parameters (e.g., light source characteristics, intensity, duration, and timing for phototherapy) lacks consensus, necessitating standardized large-scale studies to establish evidence-based treatment protocols. Evidence level for rTMS: Several randomized controlled trials in human subjects have demonstrated that high-frequency rTMS over the dorsolateral prefrontal cortex improves cognitive performance and reduces subjective fatigue following SD (133). However, optimal stimulation parameters (frequency, intensity, duration, and timing) remain to be standardized, and most studies have small sample sizes (140). Limitations: Heterogeneity in stimulation protocols; lack of long-term follow-up data; variability in individual responses.

### 7.3 Exercise

Exercise, beneficial for physical and mental health, is increasingly applied clinically to counter SD. Evidence suggests that CSD leads to changes in the hippocampus responsible for spatial memory. While 48 h of acute SD significantly impairs spatial memory in rats (assessed by the Morris water maze), mild, regular treadmill exercise can mitigate these adverse effects of acute SD on memory (141), and enhance blood glucose control in rats (142), and mitigate changes in glucose tolerance, mitochondrial function, myofibrillar protein synthesis, and circadian rhythms induced by insufficient sleep in healthy subjects (143).

### 7.4 Cognitive behavioral therapy

Cognitive Behavioral Therapy (CBT) is a multi-component, non-pharmacological approach for treating SD caused by various factors. Core elements of CBT for insomnia (CBT-I) include stimulus control, sleep restriction, relaxation techniques, and sleep hygiene education. For chronic insomnia, CBT has proven highly effective in numerous randomized controlled studies and has demonstrated better long-term efficacy than prescription sleep medications (144). CBT is effective in reducing sleep onset latency and wake after sleep onset. CBT duration typically ranges from 4 to 8 weeks. An open-label single-group trial implemented by Monika Pathania et al. showed that an online breath meditation workshop (OBMW) improved sleep and heart rate variability (HRV) in members of parliament, effectively countering stress and SD (145).

### 7.5 Dietary interventions

Chronic insomnia or SD impairs cognitive function and brain glucose metabolism. The ketogenic diet (KD) offers an alternative fuel source, with ketone bodies potentially providing metabolic benefits during SD. Wang et al. demonstrated that KDs enriched with medium-chain triglycerides (MKD) and long-chain triglycerides (LKD) prevent SD-induced cognitive deficits by inhibiting ferroptosis and improving synaptic plasticity. MKD showed stronger effects in ameliorating cognitive deficits than LKD (146). A randomized crossover clinical trial showed that, compared to a carbohydrate-based diet, a KD improved psychomotor vigilance task performance and memory continuous performance test performance in military subjects, demonstrating beneficial effects on cognitive performance, mood, and sleepiness during 36 h of sustained wakefulness (147) (Table 3).

**Table 3:** Comparison of Pharmacological and Non-Pharmacological Interventions for SD.

Intervention class	Examples	Evidence level	Effect size	Limitations/Adverse effects	Targets underlying cause or symptoms?
CNS Stimulants	Caffeine, modafinil	Multiple RCTs	Moderate	Tolerance, withdrawal, circadian disruption (caffeine); headache, nausea, potential for abuse (modafinil)	Symptomatic (maintains alertness)
Sedative-Hypnotics	Zolpidem, ramelteon	RCTs for insomnia	Small–moderate	Residual sedation, dependence risk; ramelteon has limited chronobiotic efficacy	Symptomatic (promotes sleep)
Orexin Receptor Antagonists	Suvorexant, daridorexant	RCTs for insomnia	Moderate	Daytime somnolence, potential for next-day impairment	May target underlying sleep–wake dysregulation
Traditional Chinese Medicine	Liuwei Anshen, Banxia Xiexin	Preclinical only	Not quantified	Lack of human RCT data; unknown safety profile in long-term use	Unknown (mechanistic studies suggest anti-inflammatory/chronobiotic effects)
Gut Microbiota Modulators	Probiotics, prebiotics	Preclinical predominates; limited human	Small	Strain-specific effects; heterogeneity in formulations	May target underlying gut–brain axis dysregulation
Napping	20–60 min naps	Multiple RCTs	Moderate	Sleep inertia if nap >30 min; requires opportunity	Targets underlying sleep debt
Physical Therapies	rTMS, tDCS, taVNS	RCTs (small sample)	Moderate	Optimal parameters not standardized; variable individual responses	May target underlying neural circuit dysfunction
Exercise	Aerobic, resistance	Preclinical; limited human	Small–moderate	Timing of exercise matters; not feasible during acute SD	Targets underlying metabolic and circadian disruption
Cognitive Behavioral Therapy	CBT-I	Multiple RCTs	Large for chronic insomnia	Requires a few weeks; not suitable for acute SD	Targets underlying behavioral and cognitive factors
Dietary Interventions	Ketogenic diet	Limited human RCTs	Moderate	Adherence difficulty; long-term safety unknown	May target underlying metabolic disruption

## 8 Conclusion

**Figure 1:**
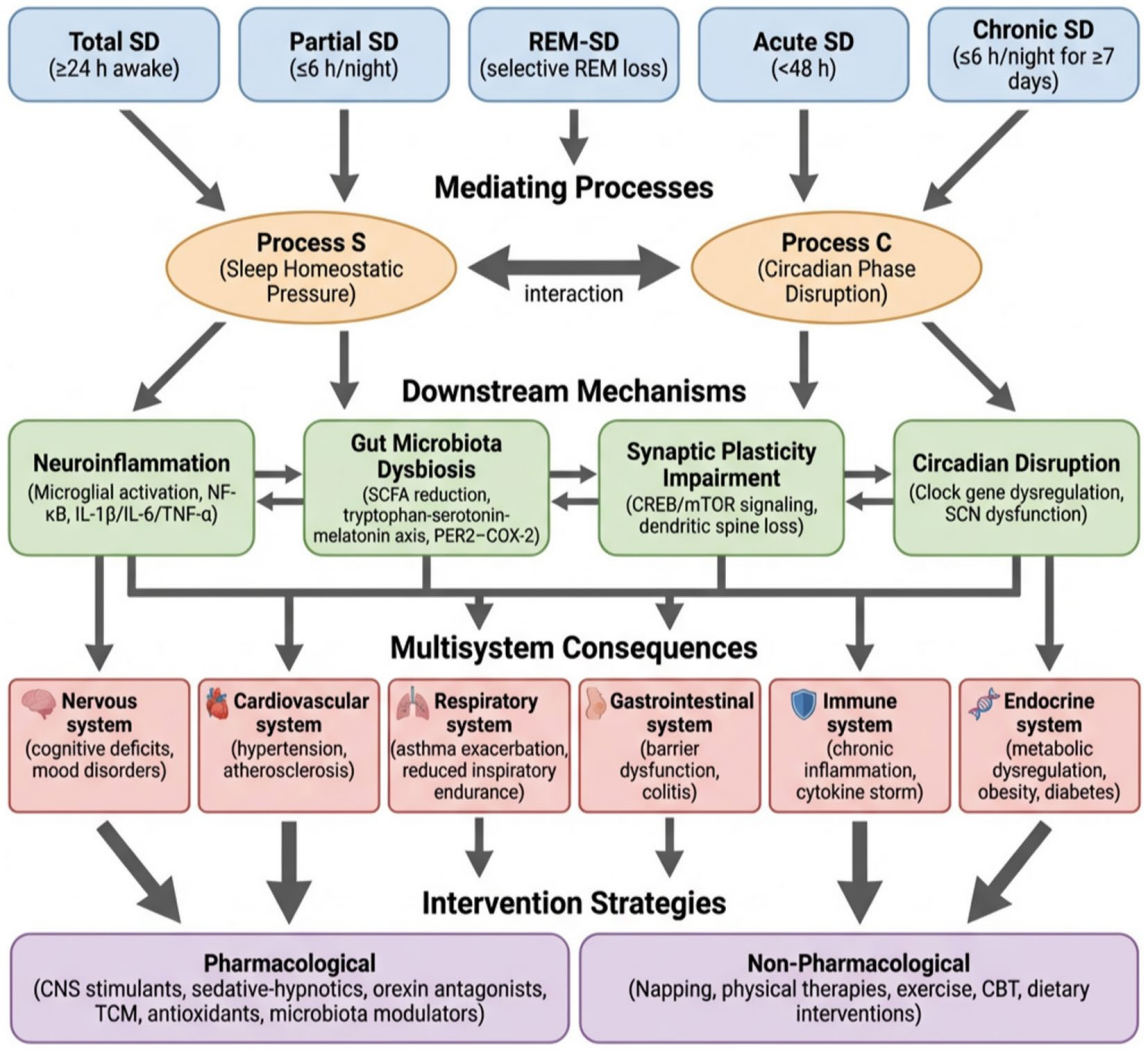
Multisystem impacts and core mechanisms of SD.

This review elaborates on the impact of SD on human health, discusses the underlying mechanisms at the molecular and cellular level from an animal model perspective, and focuses on relevant intervention strategies (Figure 1). Due to various constraints, this review cannot provide an evidence-based critique of every study but instead aims to synthesize and present the latest information from the literature on SD, its health consequences, and interventions, thereby enhancing public knowledge about SD.

The immediate response to SD is fatigue, manifested as sleepiness, slowed reactions, and listlessness. Consequently, central nervous system stimulants like dextroamphetamine, caffeine, and modafinil are clinically used to counter the negative effects of SD. While they can provide short-term symptomatic improvement, long-term use may lead to dependence or adverse reactions. Newer, safer formulations, used alone or in combination, are being explored, such as probiotic-melatonin combination therapies based on gut microbiota modulation, and non-pharmacological interventions like transcranial magnetic stimulation and phototherapy. Combined approaches, such as brief napping coupled with repetitive transcranial magnetic stimulation (rTMS), are also being investigated to offset the negative effects of SD (148).

Given the significant individual differences in tolerance to SD and in drug efficacy and side effects, future research should consider: (1) For the elderly population, studies should focus on the impact of multimorbidity and polypharmacy, recommending non-pharmacological interventions like earplugs and eye masks to limit noise and disturbance, or adjusting light intensity according to natural sleep cycles (149); (2) Pediatric research needs to focus on the long-term associations between medication and neurodevelopment, potentially establishing age-specific SD risk assessment systems. (3) For special occupational groups (e.g., healthcare workers, shift workers), SD intervention strategies should be personalized based on their specific work characteristics. However, these interventions should be implemented alongside lifestyle modifications that promote healthy sleep patterns, as the benefits of good sleep hygiene are substantial. The advancement of technologies like functional magnetic resonance imaging (fMRI) and electroencephalography (EEG) will help explore the pathogenic mechanisms of SD and provide effective interventions (150), paving the way for developing more effective treatment and prevention strategies.
